# Dismantling AI capitalism: the commons as an alternative to the power concentration of Big Tech

**DOI:** 10.1007/s00146-022-01437-8

**Published:** 2022-04-09

**Authors:** Pieter Verdegem

**Affiliations:** CAMRI, Westminster School of Media and Communication, University of Westminster, London, UK

**Keywords:** Artificial Intelligence (AI), AI capitalism, Political economy, Commodification, Extraction, Commons

## Abstract

This article discusses the political economy of AI capitalism. It considers AI as a *General Purpose Technology* (GPT) and argues we need to investigate the power concentration of Big Tech. AI capitalism is characterised by the commodification of data, data extraction and a concentration in hiring of AI talent and compute capacity. This is behind Big Tech’s unstoppable drive for growth, which leads to monopolisation and enclosure under the *winner takes all* principle. If we consider AI as a GPT—technologies that alter society’s economic and social structures—we need to come up with alternatives in terms of ownership and governance. The commons is proposed as an alternative for thinking about how to organise AI development and how to distribute the value that can be derived from it. Using the commons framework is also a way of giving society a more prominent role in the debate about what we expect from AI and how we should approach it.

## Introduction

We are at the crossroads of technological developments which are changing our economy and society. It is argued that much of our productivity and prosperity will be derived from the systems and machines we are creating (Brynjolfsson et al. 2014; Hall and Pesenti 2017). Artificial Intelligence (AI) is one of the most hyped innovations of our times. In business circles, AI is seen as a catalyst for growth, which will manifestly transform the economy (Agrawal et al. 2018; Lee 2018; McAfee and Brynjolfsson 2017). Policymakers are looking at the opportunities of AI for tackling global challenges, such as climate change (Dobbe and Whittaker 2019) or pandemics (Tzachor et al. 2020), while AI is also the subject of an arms race between the US, China and Russia to have their military forces equipped with automated weapons (Asaro 2019).

While AI is around for more than 60 years and periods of hope and optimism have been alternated with so-called *AI Winters*, it seems crucial parts of the puzzle finally have started to fall into place. The confluence of factors—the availability of powerful computing capacity, new techniques in machine/deep learning leading to more sophisticated algorithms and the growing availability of data with which to train these algorithms—enable AI to be deployed far more extensively (Elliott 2019; Hall and Pesenti 2017; Lee 2018). AI now seems ready to have a deep impact on our society and economy.

Especially, since 2015, a peak in corporate investment, a growing number of mergers and acquisitions and more intensive competitive hiring of AI talent can be noticed (Dyer-Witheford et al. 2019; Lee 2018). This is not surprising given assessments about the (future) size of AI in industry. For example, PwC (2017) predicts AI could contribute up to $15.7 trillion to the global economy by 2030. The same study states that the greatest economic gains from AI will be in China (26% boost to GDP by 2030) and North America (14.5% boost) (PwC 2017). This is not unexpected as the US and China are in an intense competition to become world leaders in AI (Lee 2018).

The industrial landscape of AI, however, is dominated by *Big Tech*, a small number of extremely powerful companies. There are only a few companies that own exponential computing power, can attract AI talent and have access to data to develop and train advanced machine/deep learning models. AI is a *General Purpose Technology* (GPT), an enabling technology that impacts on how large sections of the economy and society are organised. Because AI is a GPT, we need to analyse *AI capitalism*. In particular, we want to understand how AI capitalism is organised, what is driving its concentration of power, and its impact. Beyond this, we also need to think about alternatives that can help mitigate the negative consequences of this power concentration and make sure that society at large can benefit from the new wave of AI innovation.

This article starts with considering AI as a GPT and argues why we need to focus on power when thinking about the impact of AI. I explain the contribution of *critical political economy* (CPE) for analysing AI capitalism. CPE investigates control and ownership of communication systems and its impact on society (Hardy 2014). Using CPE as a framework, this article analyses the tendencies of concentration and monopolisation in AI capitalism. The article then considers the commons as an alternative framework for enabling that the benefits of AI can be shared with society at large.

## AI as a GPT

An important aspect of understanding AI capitalism is to consider AI as a *General Purpose Technology* (GPT) (Trajtenberg 2018). GPT are enabling technologies, meaning that they open up new opportunities, in addition to offering complete, final solutions. Other examples of GPT are the steam engine, electrification and the Internet. They have three main characteristics: (1) they are widely used; (2) they are capable of ongoing technical improvement; and (3) they enable innovation in different application sectors (Bresnahan, 210: 764). AI qualifies this definition.

Given their pervasiveness and the complementary waves of innovation they produce, GPT cause economic disruption. They affect entire economies, potentially drastically altering societies through their impact on pre-existing economic and social structures (Trajtenberg 2018). Economists study the impact of GPT in terms of the emergence of winners and losers. The *winners* are those associated with the emerging GPT, whereas the *losers* are those who cannot benefit from the unfolding GPT. However, looking at other GPT invites us to look beyond winners and losers and to consider GPT—and thus also AI—as a public utility. The importance of electricity and the Internet, for example, has opened debates about the need of regulation, and the decision to not merely leaving these technological developments over to the market alone, or at least to have some intervention from society in it. This is particularly important given the times we live in: during the pandemic, we all have witnessed the crucial role of digital platforms in everyday life. As such, we need to be aware that AI can facilitate a further polarisation of already unequal societies (Crawford 2021; Dyer-Witheford et al. 2019; Lee 2018).

In any case, considering AI as a GPT and not just a digital technology that is owned and used by private entities but one that has broad impact on society, opens up new questions about how to conceptualise AI capitalism. This is where the work of Kate Crawford comes into place. In her book *Atlas of AI,* Crawford (2021) offers a comprehensive and nuanced understanding of AI. According to her, AI simultaneously refers to *technical approaches*, *social practices* and *industrial infrastructures* (Crawford 2021: 8–9).

First, AI refers to technical approaches. Advancements in *machine learning* (ML) have been the most powerful contributor to the development of AI in the past two decades (Asaro 2019). ML is a paradigm that allows programs to automatically improve their performance on a particular task by learning from vast amounts of data (Russell and Norvig 2016; Lee 2018). It is based on statistical patterns and correlation in large data sets, starting to be used in the late 1980s–early 1990s. Earlier versions of machine intelligence—e.g., expert systems—were primarily rules-based, making use of symbolic logic and involving human experts generating instructions codified as algorithms (Agrawal et al. 2018). The problem was that they could not cope with the complexity of most applications. Unlike expert systems, powerful ML algorithms learn from the ground up, not from humans but from data (Alpaydin 2017). The rise of ML can be explained by more powerful and reliable computing infrastructure, which has made possible the development of systems driven by real-world data (Lee 2018). The availability of significant amounts of data further enables the development of learning algorithms that derive solutions using statistical methods. *Deep learning* (DL) and *neural networks* (NN) are the driving forces behind more recent developments in ML. In the early 2000s, ML pioneer Geoffrey Hinton (LeCun et al. 2015) demonstrated the power of DL neural networks: this allows automatically processing of unlabelled data, which has led to more effective applications of AI that we are now using every day (e.g., online services).

Second, the *social practices* of AI refer to the classification systems, developed by humans, which are behind algorithms, ML/DL models and AI systems. Crucial questions that we need to ask here are: Who is involved in developing these classification systems? Who decides what classifications are used? and; What do they look like? Ultimately, these are political (power) questions about inclusion and representation (Crawford 2021). Important challenges exist around AI, bias, fairness and discrimination (Costanza-Chock 2018). Questions about how representative these classification systems are, are crucial in this. How to avoid bias and support inclusion in AI systems are important political issues that urgently need to be addressed (Brevini and Pasquale 2020).

Last, the *industrial infrastructures* of AI refer to the computing power, algorithms and data sets that are the source of knowledge and production. This infrastructure not only entails the possibilities of collecting vast amounts of data—which are needed to train algorithms—but also the computational power necessary to develop and perform ML and DL models. Few companies have access to the required data sets, possess the necessary computational power to run ML/DL and are able to attract the brightest AI scientists, which means we are witnessing a concentrated industrial AI infrastructure, leading to AI oligopolies/monopolies (Dyer-Witheford et al. 2019; Riedl 2020). This gives a lot of power in the hands of a small number of corporations (Montes and Goertzel 2019)  and is why we need to scrutinise economic power within AI capitalism.

Offering an encompassing view of AI capitalism, is important: we need to be aware of how material AI is and that its production is based on natural resources, human labour and industrial infrastructures. Looking at the broader picture of change within technologies, beliefs and infrastructures simultaneously, however, also risks overlooking the issues of a concentration of power. To deal with this, we need to go back to political economy as this is the framework that puts power at the centre of its analysis. Political economy is particularly interested in the relationship between techno-economic systems and their impact of the broader societal structure (McChesney 2000). The industrial infrastructures of AI also contribute to a concentration of power, which has not only an impact on the social practices of AI but also how its technological development will happen in the future, which explains the importance of this perspective.

## The political economy of AI capitalism

### Critical political economy as a framework

Political economy focuses on how societies are organised and controlled, and therefore, power is a focal point of attention. Rooted in the 1970s, *political economy of communication* (PEC) is fundamentally interested in studying the relationship between media and communication systems and the broader society. British scholars Murdock and Golding (1973), some of the founders of the discipline, argued that PEC analyses media and communication as commodities produced by capitalist economies. Political economy is particularly interested in how economic factors influence politics and social relationships (McChesney 2000). It is concerned with who has the power to make decisions about media and communication systems and who benefits from these decisions. In other words, PEC investigates how power relations work within and around media and communication systems.

*Critical political economy* (CPE) has developed within the tradition of PEC and is recognised as a distinct framework (Hardy 2014). It refers to approaches that emphasise the unequal distribution of power. As such, CPE is critical of contexts and practices within which inequalities are sustained and reproduced. CPE is influenced by but not limited to Marxian political economy. The latter tradition has provided a historical analysis of capitalism, whereby specific attention is given to the forces and relations of production, commodification, the production of surplus value, class and social struggles. CPE focuses on issues relating to the growing concentration and privatisation of media and communication industries, as well as the impact of commodification and globalisation. CPE analyses the structure and consequences of ownership regimes but also the relationship between government policies and industry. Last, CPE also focuses on challenging the dominant ideology that legitimises the capitalist system.

### The emergence of AI capitalism

In the aftermath of the global financial and economic crisis of 2007–2008, two separate albeit related developments contributed to an environment in which AI capitalism could emerge: a changing political context and a technological transformation.

First, the broader political context has changed by what Standing (2016) calls a *global transformation*. Essentially, he refers to a shift in the political context from neoliberalism to rentier capitalism. Neoliberalism promotes free markets and includes other dynamics and characteristics, such as commodification, privatisation and labour market reregulation (not deregulation) in favour of flexibility in terms of labour and capital (Harvey 2005). Rentier capitalism, on the other hand, refers to a system in which efforts are made to enlarge one’s existing share of wealth without actually contributing to the creation of new wealth (Christophers 2020; Standing 2016). It constitutes a model of monopolistic rent-seeking, which often leads to markets dominated by a small number of extremely powerful multinationals (Birch 2020).

Second, at the same time we are witnessing a *technological transformation*, which increasingly dominates and transforms the capitalist system. The *technological* element refers to the Internet and other related digital technologies that emerged since the early 2000s (social media, the Internet of Things and AI). Other scholars have framed this *digital capitalism* (Schiller 2000), *informational capitalism* (Fuchs 2010), *platform capitalism* (Srnicek 2017), *data capitalism* (Sadowski 2019; West 2019) and *AI capitalism* (Dyer-Witheford et al. 2019).^1^

In the next paragraphs, I discuss the main building blocks of what constitutes AI capitalism and identify the problems within it. I first discuss the problems related to data (commodification and extraction) and then elaborate on the concentration of power within the hiring of AI talent and computing infrastructure.

### The commodification of data

*Commodification* is a central concept in CPE and refers to the processes, whereby online and offline objects, activities, ideas and emotions are transformed into tradable commodities, transforming use value into exchange value (Hardy 2014). In the context of AI capitalism, commodification is closely linked to *datafication*. The latter concept refers to the ability to render into data many aspects of the world that have never been quantified before (Cukier and Mayer-Schoenberger 2013). Our social relationships, communication patterns, shopping behaviour, etc. are transformed into digital data (Couldry and Mejias 2019), which is an essential characteristic of the attention economy (Wu 2017).

In AI capitalism, the interplay between data and digital platforms is important. *Platforms* are intermediaries that invite different types of users—producers and suppliers, consumers, advertisers, app developers, etc.—to engage and interact via their digital infrastructure (Srnicek, 2017; Van Dijck et al. 2018). Platforms are ideally positioned to function as a data broker: central in their business model is the possibility to capture, extract and analyse the data produced by the interactions on the platform (Crain 2018; West 2019). Using this extracted data as well as the skills workers gained when analysing it, made platform companies the leaders in the digital economy; working with data has become ever more important for gaining a competitive advantage (Srnicek 2018).

What connects data and platforms are *network effects*. Network effects mean that the value of the network is determined by its size (Katz and Shapiro 1985). Platforms thus become more valuable as more users join it. Engagement and interaction are only possible if there are active users on platforms. Generating network effects is thus a key strategic focus for platforms (Srnicek 2017). The power of network effects goes hand in hand with the availability of data: this combination further strengthens the leading position of already powerful data companies (Srnicek 2018). Data-driven network effects entail that more users active on a certain platform, means more possibilities for data collection, analysis and extraction. Consequently, this results in more opportunities to use that data for improving the features and services offered by the platform. Better services open up the possibility to attract more users. A similar positive *data feedback loop* exists for AI too: better access to data means more opportunities to train ML models and better AI also results in better services and more users (Lee 2018; Srnicek 2018; Varian 2018).

### Data extraction

A second key characteristic of AI capitalism is the centrality of *data extraction*. We can conceptualise data as two distinct economic forms: First, data is a raw material—constant capital—which is necessary for the production of commodities (Crain 2018). AI companies use data such as raw materials to produce various informational goods and services, what Shoshana Zuboff (2019) calls *prediction products*. Data sets are an essential resource to train ML/DL models. Second, data itself is a commodity, the product of the digital labour of people engaging with applications and services offered by platforms.

While data is often considered as a raw material or a commodity, it makes sense to conceptualise it as a form of capital too. This is part of a broader discussion about how value is generated in the contemporary economy (Arvidsson and Colleoni 2012; Mazzucato 2018), particularly how value is derived from data and what normative aspects are relevant in the context of data collection and extraction (Couldry and Mejias 2019; Mezzadra and Neilson 2017; Zuboff 2019). Sadowski (2019) argues that treating data as capital allows for a more nuanced and detailed understanding of how AI capitalism functions and is organised.

What is the problem with using data to create value, as a resource to develop and optimise AI systems? Mazzucato (2018) analyses contemporary capitalism and highlights the critique that it rewards *rent seekers* over true *value creators*. Their rent seeking is based on overcharging prices, undercutting competition—by exploiting particular advantages, e.g., labour, or using a monopoly advantage. Where *value creation* refers to the use of different types of resources to produce new goods and services, *value extraction* is defined as “*activities focused on moving around existing resources and outputs, and gaining disproportionally from the ensuing trade*” (Mazzucato 2018: 6). Data extraction is a particular type of value extraction. Sadowski (2019: 9) defines *data extraction* as: “data is taken without meaningful consent and fair compensation for the producers and sources of data”*.* Evgeny Morozov (2018) follows a similar line of thinking and has coined *data extractivism* to refer to practices of tech giants launching products not for the revenue but for the data, which is afterwards monetised through different products and services (see also Couldry and Mejias 2019). It is clear we must scrutinise what the consequences are of data commodification and extraction in AI capitalism as well as considering alternatives.

### AI talent

AI capitalism is dominated by the so-called *Big Tech*; tech giants that dominate and control the market. These companies are often referred to by the acronyms *GAFAM* and *BAT* (Kaplan and Haenlein 2020; Verdegem 2022). GAFAM refers to US-based companies and includes Google (Alphabet), Apple, Facebook (Meta), Amazon and Microsoft. BAT refers to tech companies in China, including Baidu, Alibaba and Tencent.

Especially, since 2015, the leading tech companies have intensified the competition for hiring AI talent, i.e., the computer science experts who are at the forefront of developments in machine/deep learning (CB Insights 2021). They mainly do this by acquiring AI startups and only face competition from blockchain companies and the military.

Google (Alphabet) purchasing the UK-based startup DeepMind (founded in 2010), the company that developed the DL models behind the famous victory of AlphaGo over Lee Sedol, is one of the most famous examples of acquisitions in the field of AI. Since then, DeepMind has become one of the world’s leading AI companies. All of the mentioned Big Tech companies have been very active in taking over startup companies with the purpose of acquiring AI expertise and talent. According to CB Insights (2021), Apple has made 29 AI acquisitions since 2010, Google (Alphabet) 15, Microsoft 13, Facebook (Meta) 12 and Amazon 7. A similar pattern is followed by Big Tech in China (Lee 2018).

While companies, such as IBM, Intel, Salesforce and NVIDIA—active in hardware (semiconductors) and software development—also try to establish themselves in the growing market and engage in take-overs of AI startups, the fiercest competition is happening at the level of smaller companies and/or startups. These companies are positioning themselves to either trying to occupy a profitable AI niche (which they hope might develop into a larger segment of the market) or to be taken over by one of the giants (Lee 2018). The problem this intense competition for AI talent creates is that it leads to a divide between the developers of ML/DL models who are hired by Big Tech and who can ask enormous salaries (Metz 2017), and the rest of computer scientists and other groups in society who are paid less or are even exploited for doing the work in the hidden infrastructure of AI (Crawford 2021; Altenried 2020). Hence, the need for alternatives becomes more prominent.

### AI compute capacity

AI capitalism is not only determined by data commodification/extraction and the fierce competition over AI talent; another aspect that is crucial for AI dominance is computing power (Ahmed and Wahed 2020; Srnicek 2019). AI compute capacity refers to hardware and software engineered to support the development of AI applications. It includes large data centres, supercomputers and cloud providers. Having the most powerful and performant AI compute capacity is necessary for dominating the AI market.

Amazon (Amazon Web Services—AWS, launched in 2002) and Microsoft (Azure, launched in 2008) have traditionally been dominant in the market of cloud computing. More recently, especially since 2015, there have been major investments in data centres, supercomputers and cloud computing (Dyer-Witheford et al. 2019; Srnicek 2019). This can be explained by the fact that more businesses—beyond tech—became data-driven and need this infrastructure to process the data being collected as part of new services and business models. Still, the biggest investments in AI compute capacity are made by Big Tech. Companies such as Alibaba (Aliyun), Baidu (Wangpan), Google (Google Cloud) and Tencent (Tencent Cloud) have been investing massively in cloud computing with the goal to increase their market share (Verdegem 2022).

There is a clear explanation, relevant to our understanding of AI capitalism, why Big Tech has stepped up its investment in AI compute capacity. For making AI applications a reality—such as self-driving cars or AI systems used in the medical sector—an upgraded technical infrastructure is crucial. Performant computing infrastructure is an absolute key issue in terms of security, reliability, and speed. The roll-out of new AI systems diminishes the tolerance towards network latency and security issues. The problem is that only big companies, which have a lot of capital at their disposal, can make these investments. In addition, it is only Big Tech that has the resources to upgrade their compute capacity while simultaneously being able to collect data to train ML/DL models and to hire the specialised AI talent to work on these models. Ahmed and Wahed (2020) have documented the unequal access to compute capacity and argue that this creates divides between big tech corporations and elite universities who squeeze other companies and the computer departments of medium and smaller universities out of the field. Srnicek (2019) also points at the power of AI behemoths, who become global rentiers through their AI infrastructure: smaller companies are dependent on the hardware of Big Tech to make advancements in AI, whereas the leading AI companies can keep control over what is happening on their infrastructure. This power concentration is thus also potentially weakening the development of AI itself.

### The AI industrial landscape: a concentration of power

The AI industrial landscape is dominated by a small number of companies. Table 1 gives an overview of how much the value of each of these giants (GAFAM and BAT) has increased in the last decade. This table illustrates how the commodification of data, in combination with data extraction, made these companies extremely profitable. The top ten of most valuable companies in the world is now dominated by AI companies (Statista 2020). Their drive for expansion resulted in intense concentration, where each one of them has achieved a highly dominant position in the market (Kaplan and Haenlein 2020; Montes and Goertzel 2019). For example, Google obtained an (almost) monopoly over online search, Amazon and Alibaba over e-commerce and Facebook and Tencent over the US and Chinese markets of social networking.

**Table 1:** Value* of the world's leading AI companies

Company	Year founded	2010	2015	2019	2020	2021
Google/Alphabet	1998/2015	168	387	900	841	1577
Apple	1976	238	750	940	1227	2194
Facebook/Meta	2004/2021		230	556	521	922
Amazon	1994	63	197	954	1154	1749
Microsoft	1975	270	387	995	1320	1899
Baidu	2000	22	77	58	33	73
Alibaba	1999		212	484	540	626
Tencent	1998	37	198	473	510	764

Source: Market Cap in $bn (End of April of every year) (ycharts.com, 2021)

^*^Expressed as Market Cap(italisation)—How much a company is worth, determined by the stock market

The drive for growth of AI companies demonstrates that the problem of monopolisation is real: it creates a massive power concentration in the hands of a few players. The AI giants dominate the field and aggressively acquire potential competitors. We face a situation, where there are only a few AI behemoths, who own the expensive computational infrastructure, have access to vast amounts of data to train ML and DL models and can attract the highly skilled AI talent to develop new systems and services. The economic power, expressed in their rapidly increasing value, highlights the point of AI as a GPT creating winners and losers (Trajtenberg 2018). The AI giants are definitely the *winners* in this context.

Table 1 also illustrates that the COVID-19 pandemic has only accelerated the tendency of expansion and monopolisation. The value of the AI giants has grown exponentially since the start of the pandemic, as we are all massively dependent on their digital services. One of the risks of this domination is that these companies alone have the economic power to make political decisions about how AI is developed, how it is used and what its impact will be. It is an illustration of the *winner-take-all scenario* (Srnicek 2017).

What is a source of concern is that the AI giants follow a strategy of *enclosure*, with the objective to maintaining their leading position and safeguarding their growth and profit. Enclosure entails that—after having achieved a monopolistic position—these AI companies move to control access to their data and limit the ability of users to switch to competitors, thereby *enclosin*g more and more of the digital world within their private sphere (Couldry and Mejias 2019; Morozov 2018).

The enclosure by AI capitalism is clearly illustrated by OpenAI. Originally founded as a non-profit organisation, which would collaborate with other institutions and researchers and make their research open to the public, OpenAI is now dominated by corporate investors, including Microsoft, and is considered as one of the biggest competitors of DeepMind.

In this context, it becomes clear that we—as a society—need to reflect on this situation and come up with alternatives so to avoid we end up as *losers*, under the control of the winners, the AI giants. Only criticising the problems of AI capitalism, however, will not be enough. As a society, we need to start imagining what alternatives could challenge the power concentration of Big Tech. *Critical political economy* offers a framework to inquire about this.

## Imagining alternatives

### Introducing the commons

In most simple terms, the commons are the natural and cultural resources that are accessible to all members of society. What is typical about them is that they are held in common, instead of being owned privately (Bollier 2014). Public debate about the commons has become more mainstream due to environmental degradation and has been popularised—amongst others—by the first female winner (2009) of the Nobel Memorial Prize in Economics, Elinor Ostrom. Her work includes *Governing the Commons* (Ostrom 1990), in which she refutes the *Tragedy of the Commons* thesis (Hardin 1968). She has inspired thinking about the design and organisation of cooperative alternatives beyond markets and states.

Ostrom, together with her colleague Hess, has also worked on extending the debate about commons to knowledge. Hess and Ostrom (2007) approached knowledge as a complex ecosystem that operates as a common, similar to what Benkler (2006) theorised as *commons-based peer production*. In a similar vein, others have been working on the concept of *digital commons*, which refers to the communal ownership and distribution of informational resources and technology (Birkinbine 2018). Taking the ideas of knowledge and digital commons together opens up opportunities to inquire about alternative structures for AI ownership and governance.

We are confronted with intense competition and concentration in AI capitalism, a situation similar to what has been labelled the *enclosure of the commons*. According to Bollier (2014), the latter refers to a situation in which corporate interests appropriate our shared wealth and turn it into expensive private commodities. This is happening also in the digital sphere, whereby platforms control access to data and increasingly enclose the digital world within their private sphere. Resisting this—by pushing for alternatives—can be done by stressing the importance of data and AI as public goods, produced by society and its members (Taylor 2016; Viljoen 2021). The important task then is to explore how the commons can be reclaimed.

While thinking about the commons has its roots in radical political economy, there is a disagreement about what the end goal of its project should be. Some position the commons as an emergent value system that has the potential to transform or even replace capitalism (Broumas 2017), while others perceive the value of the commons in how it can respond to the excesses and exploitative tendencies of capitalism (De Angelis 2017). As such, the commons are not per se a replacement of capitalism but rather something that can co-exist and couple with capital circuits through the commodity firm.

### Data commons

How can we think about the commons in the context of AI capitalism? First of all, we need to conceptualise the data commons. Bria (2018) defines *data commons* as a shared resource that enables citizens to contribute, access and use data as a common good, without or with limited intellectual property restrictions. Instead of considering data as a commodity or capital (Sadowski 2019), it can be thought of as a collective resource (Viljoen 2021). As such, it can empower citizens and help them solve shared—common—problems.

The bigger picture of negotiation and agreements around data commons is part of calls for a *New Deal on Data* (Bria 2018). A report of the *Decode project*^2^ explains what such a deal on data could entail (Bass et al. 2018): First, there is a need to push for more transparency, accountability and trust in data projects; Second, individuals should be given more control and people should be empowered to decide how their data is collected and used; and, Last, it should be an important ambition to unlock more value of data as a common good while protecting people’s privacy and encouraging fair terms of use.

Of course, there are questions how to practically organise this. A lot of inspiring work on the data commons proposes solutions in terms of data infrastructure and data trusts (Coyle 2020). A new data infrastructure should help dealing with institutional and regulatory aspects of how data can be shared, what standards and policies should be set up and which organisations and communities should be involved in contributing to and maintaining this data infrastructure. One approach for an innovative data infrastructure has been developed and trialled in several countries: data trusts. *Data trusts* can exist in many forms and models but the general principle is that they sit between an individual generating data and a company or institution wanting to use that data (Delacroix and Lawrence 2019). In this system, control over data is transferred to a third party, which can use the data for pre-defined purposes. Data trusts can use data from different sources and allow to steward data use for all. Important in its governance is data solidarity, meaning that corporate and public data shareholders share the benefits and risks of data access and production (Bunz and Vrikki 2022). Coming up with a system for sharing and giving access to data does not only benefit society; it is also necessary for AI innovation (Hall and Pesenti 2017).

### Compute capacity for the commons

Compute capacity is the second element of a commons approach, as an alternative to the power concentration of AI capitalism. Some even position computing infrastructure as part of the *data commons* itself (Grossman et al. 2016). I discussed already how crucial computing power is for the development of AI. Only Big Tech (and some elite universities) have the resources to upgrade their infrastructure—contributing to an *AI compute divide* (Ahmed and Wahed 2020)—while leading AI companies collect rent from and keep control over what is happening on their compute infrastructure (Srnicek 2019). As an alternative, investments in common/public compute capacity could help society becoming less dependent on the private infrastructure of Big Tech.

While the corporate sector often claims that public investment stifles innovation, (Mazzucato 2013) debunks this myth and actually argues that the radical technologies behind, for example, the iPhone (e.g., GPS, touch screen display and Siri) were all backed by government funding. Another example is Google’s search algorithm, which was publicly funded through the National Science Foundation (NSF).

The first supercomputers were used by universities (in the US and the UK) and governments should consider pooling (more) resources to invest in (national or international) compute capacity that will drive the future of AI. Common investment in AI compute capacity will also help to democratise AI (Riedl 2020), meaning that more people and organisations can be involved in developing AI systems. This is particularly relevant for quantum computing, which is considered crucial for revolutionary breakthroughs in the future of AI—the so-called *quantum AI* (Taylor 2020). Public/common investment in computing infrastructure could also mean a de-commodification of compute capacity and create a new public service that can be made available to society, accessible to different organisations, companies and interest groups.

### A commons approach to AI human capital

While not often considered as part of the data commons, an argument can be made about common investment in AI human capital too. Having an upgraded computer infrastructure is one thing, AI human capital—the AI talent and human resources that are necessary to develop AI innovations—is as important.

Given the high level of specialisation, success in research on machine/deep learning is dependent on people who have accumulated large expertise through formal training (e.g., PhD) or years of applied work (Ahmed and Hamed 2020). As a result, there is a growing gap between the increasing demand for AI expertise and the limited supply, resulting in a talent scarcity (Metz 2017).

A commons approach to AI human capital would, for example, include to provide more funding for public IT services and universities allowing them, respectively, to reduce outsourcing and facilitate more research labs to keep their faculty members instead of being recruited by larger, corporate, organisations with deep pockets.

### Towards an alternative political economy of AI

Investment in public infrastructure and resources can support commons-based economies and models of organisation which allow to depart from an incentive structure focused on value creation rather than value extraction (Kostakis and Bauwens 2014). However, this depends on new regimes in terms of ownership, control and governance.

First, a central aspect of envisioning an alternative political economy of AI is rethinking ownership. Regulation is often proposed as a strategy to limit the market/monopoly power of Big Tech (Posner and Weyl 2018). Competition and antitrust law, for example, could be used to break up the AI/tech giants. However, such a strategy might be counter-productive, as the power of, for example, social media platforms is that they connect everyone in society. Common ownership might be an alternative approach that could be more productive (Kostakis and Bauwens 2014). There is a solid case for placing the technologies producing AI in public and collective ownership. It would mean that communities have more control over how AI is produced and how the public can benefit from its services. The end goal is to have a digital infrastructure that is available to and provides advantages for a broad range of stakeholders in society, not just the AI behemoths.

Second, related to ownership is the aspect of promoting common governance. The goal here is the democratisation of AI and this requires the decentralisation of power, back in the hands of the public (Posner and Weyl 2018; Riedl 2020). If we consider AI as a GPT, which will alter the structures of society, we need to make sure there is democratic oversight and control. After all, we have installed regulators that have the power to protect the interests of citizens in other sectors, such as postal services, electricity, broadcasting and telecommunication. The services provided by AI are so crucial in everyday life, making it necessary that society has a greater say about it.

Inspiration for alternative structures in terms of ownership, control and governance can be found in the *platform cooperativism* model (Scholz 2017), which allows involvement from multiple stakeholders in the ownership, development and management of platforms.

Finally, we need to come up with a new vocabulary when thinking about AI systems and how they deliver benefits to society. Instead of corporate discourses portraying AI as *Tech for Good*, boosting innovation and entrepreneurship, it makes sense to perceive AI infrastructures as a computational utility, subject to democratic control (Mosco 2017). Dyer-Witheford and colleagues (2019) elaborate on this and push for considering AI as a *communal utility*. This means that communities and workers should be involved in determining what sort of work should or should not be automated, and thus call for a genuine determination by the *general intellect* in the design of AI. In this general intellect, collective cooperation and knowledge become a source of value (Terranova 2000). The proposed principles of common ownership and governance should be central in developing AI as a communal utility.

## Concluding remarks

This article analyses AI capitalism. I discuss the contradictions between visions of AI as a *General Purpose Technology* (GPT) generating benefits for society at large and the reality of AI capitalism, characterised by commodification, extraction and a power concentration. These aspects are behind the unstoppable expansion of tech platforms and monopolisation in the field of AI. This leads to a winner-take-all scenario, in which AI giants follow a strategy of enclosure: controlling access to data, talent and compute capacity and enclosing more of the world within their private sphere (Couldry and Mejias 2019). As a result, few players have the power to make decisions about how AI is developed and used and what its impact on society will be. It prevents the culture of AI (Elliott 2019) being accessible for all (Verdegem 2021).

Analysing the political economy of AI makes clear we need to rethink AI ownership and governance, and how data but also human resources and computing infrastructure can be shared and made accessible to a broader range of stakeholders in society. To challenge the power concentration of Big Tech, we need to come up with alternatives.

In this article, I propose the framework of the commons for helping to imagine alternatives in the context of AI capitalism. The commons is an approach that allows us to rethink collective ownership and governance regimes and empower more groups in society to have control over and a say in the development of AI and how the benefits of AI can be shared with society at large. Instead of the GPT AI resulting in Big Tech as *winners* and most of us *losers*, a commons approach allows more groups in society to be among the winners of AI innovation.

The movement towards data and AI commons should be combined with more general initiatives to push back against the commodification of and extraction in everyday life. Establishing a more equal and inclusive society depends on the democratisation of tech and the involvement of different types of stakeholders (Posner and Weyl 2018). Citizens, civil society and other organisations should have a more prominent role in the development and management of AI, which could take form along the lines of the multi-stakeholder cooperative model (Standing 2019). The latter refers to a model of organisation (co-ops) that allow for governance by representatives of different stakeholder groups within a certain organisation.

## Footnotes

1 Dyer-Witheford et al. (2019: 50–51) make the distinction between *actually-existing AI capitalism* and *AI capitalism*. They consider these different phases within *cybernetic capitalism*, where the former refers to *narrow AI*, driven by big data, the cloud, platforms, sensors, etc. and the latter refers to increasingly advanced and autonomous AI. In my approach, *AI capitalism* is different in comparison to platform or data capitalism: it is a new logic of commodification in how capital operates but also how it transforms labour and production.

2 The *Decode project* is funded by the European Commission and focuses on alternative approaches on data and personal information management. More information via: www.decodeproject.eu.
